# COVID-19 and chronic liver disease: results from the 1219 patients French registry

**DOI:** 10.1038/s41598-025-14213-7

**Published:** 2025-10-07

**Authors:** L. Blaise, F. Lebossé, C. Costentin, S. Si Ahmed, A. Heurgué, H. Fontaine, M. Meszaros, S. Radenne, C. Vanlemmens, A. Landrieux, C. Bouzbib, H. Barraud, E. Bardou-Jacquet, O. Chazouillères, M. Latournerie, I. Rosa, R. Anty, M. Gelu-Simeon, M. Khaldi, G. Amaddeo, C. Lemaitre, B. Bernard-Chabert, L. Moga, D. Roulot-Marullo, L. Elkrief, J. Boursier, A. Plessier, C. Bureau, G.-P. Pageaux, A. J. Rémy, P. Sultanik, V. de Ledinghen, N. Reboux, F. Texier, J.-B. Hiriart, H. Montialoux, S. de Montigny, M. Rudler, N. Williet, Z. Talib, B. Mboup, M. Bourlière, E. Vicaut, N. Ganne-Carrié, J. Dumortier

**Affiliations:** 1 CHU Avicenne, AP-HP, Bobigny, France; 2 CHU La Croix Rousse, HCL, Lyon, France; 3 CHU La Tronche, Grenoble, France; 4 Hôpital Saint Joseph, Marseille, France; 5 CHU Robert Debré, Reims, France; 6 CHU Cochin, AP-HP, Paris, France; 7 CHU Saint Eloi, Montpellier, France; 8 CHRU Jean Minjoz, Besançon, France; 9 CHU Beaujon, AP-HP, Clichy, France; 10 CHU La Pitié-Salpêtrière, AP-HP, Paris, France; 11 CHU Trousseau, Tours, France; 12 CHU Pontchaillou, Rennes, France; 13 CHU Saint Antoine, AP-HP, Sorbonne University, Paris, France; 14 CHU François Mitterrand, Dijon, France; 15 Centre Hospitalier Intercommunal, Créteil, France; 16 CHU L’Archet, Nice, France; 17 CHU de La Guadeloupe, Pointe-À-Pitre, France; 18 CHU Claude Huriez, Lille, France; 19 CHU Henri Mondor, Créteil, France; 20 Hôpital Jacques Monod, Le Havre, France; 21 CHU Angers, Angers, France; 22 CHU Rangueil, Toulouse, France; 23 Centre Hospitalier de Perpignan, Perpignan, France; 24 CHU Haut-Lévêque, Bordeaux, France; 25 CHU La Cavale Blanche, Brest, France; 26 Clinique Chirurgicale de La Loire, Saumur, France; 27 CHU Charles Nicolle, Rouen, France; 28 Hôpital Edmond Garçin, Aubagne, France; 29 CHU Hôpital Nord, Saint-Etienne, France; 30 Unité de Recherche Clinique, CHU Lariboisière-Fernand Widal, Paris, France; 31 INSERM UMR 1252 IRD SESSTIM Aix Marseille Université, Marseille, France; 32 Université Sorbonne Paris Nord, Bobigny, France; 33 INSERM UMR 1138, Centre de Cordeliers, Paris, France; 34 CHU Edouard Herriot, HCL, Lyon, France; 35 Service d’hépatologie, Hôpital Avicenne, 125 rue de Stalingrad, Bobigny, 93000 France

**Keywords:** Cirrhosis, Chronic liver disease, COVID-19, SARS-Cov2, Immunosuppression, Transplantation, Hepatology, Predictive markers, Risk factors

## Abstract

The deleterious impact of Coronavirus SARS CoV-2 related Disease (COVID-19) in patients with chronic liver disease (CLD) has been previously described. We report here data from the largest French “real-life” cohort. Patients with CLD regardless of etiology and liver transplant recipients who developed COVID-19 confirmed by a positive PCR and/or an evocative chest CT scan were included. The primary outcome was 30-days mortality. Prognostic factors were analyzed using both uni- and multivariate models in subgroups of transplanted and non-transplanted patients. Between August, 2020, and December, 2021, 1219 patients were included, mostly men (n = 754), median age 61 years, 477 patients with advanced CLD (decompensated (Child–Pugh B or C) in 164 patients); 366 patients were immunocompromised, including 271 organ transplant recipients. Hospitalization in intensive care unit was required in 11% of cases. The median follow-up was 68 days. The overall 30-days mortality was 13% (159 deaths, 62% related to extra-hepatic causes). Among transplant recipients, age was the only independent prognostic factor. In the non-transplanted population, the independent prognostic factors were advanced liver fibrosis (F3 or F4, HR 2.5), obesity (HR 1.56) and age (HR 1.03), whereas immunosuppression was not. Within the subgroup of patients with advanced CLD, decompensation (Child–Pugh B or C) was an independent predictor of mortality (HR 3.8). In conclusion, our results highlight the increased vulnerability of patients with advanced CLD to COVID-19, particularly those with decompensated disease. Conversely, they confirm the absence of excess mortality related to immunosuppression, particularly in organ transplant recipients. **Clinical trial registration:** Clinical trial (NCT 04,375,670).

## Introduction

The coronavirus disease 2019 (COVID-19) Pandemic that has evolved since the end of 2019, caused by severe acute respiratory syndrome coronavirus 2 (SARS-CoV-2), has been responsible for more than 7 million deaths worldwide, including 168 000 in France. Chronic liver diseases (CLD) in the broadest sense includes pathologies of widely varying severity, and cirrhosis is implicated in 1.32 million annual deaths worldwide^1^. Several international cohorts have demonstrated the negative impact of CLD on COVID-19 prognosis^2–6^. In addition, a large French retrospective study based on the Hospital Discharge database (Programme de Médicalisation des Systèmes d’Information) emphasized that therapeutic effort limitation may have contributed to COVID-19-related death in French residents with a liver-related complication or an alcohol use disorder^6^. A large study also showed an increase in liver-related mortality during the COVID-19 period in the United States^7^. In addition, liver test abnormalities during SARS-CoV-2 infection were very common (20–65% depending on the study) and were associated with more severe SARS-CoV-2 infection^8,9^. The systemic inflammatory response induced by the virus would also be deregulated because of the immune dysfunction secondary to cirrhosis^5^. The impact of COVID-19 on liver transplant recipients has been assessed and no over-mortality was showed^10,11^.

Metabolic syndrome and excessive alcohol consumption are compounding factors associated to COVID-19 mortality^12,13^.

In this context, the French Liver Society (Association Française pour l’Etude du Foie-AFEF) has set up a national multicentric registry of patients followed for CLD infected by SARS-CoV-2 in order to describe the characteristics of their underlying liver pathologies and their evolution after infection by SARS-CoV-2 and to identify possible risk factors of death, focusing particularly on the roles of fibrosis and immunosuppression.

## Methods

### Data source

The COVID19-FOIE National Observatory is a French multicenter registry. The registry has been approved and validated by ethics committee (Comité d’Evaluation de l’Ethique des projets de Recherche Biomédicale (CEERB IRB 00,006,477), in accordance with reference method MR-004 of the Commission Nationale de l’Informatique et des Libertés.

This study was registered in the ClinicalTrials.gov database (NCT 04,375,670). As this was a noninterventional study, patients were informed and a note of nonobjection was distributed to each patient included, without the need for written consent. We confirm that all methods were carried out in accordance with relevant guidelines and regulations.

Between August 5, 2020, and December 31, 2021, patients meeting the following criteria were enrolled in the study through an ambispective approach (both retrospectively and prospectively): i) age over than 18 years; ii) previous established diagnosis of CLD of any cause and severity previously diagnosed and followed by one of the hepatologists involved in the study; iii) infection with SARS-CoV-2 diagnosed accordingly to current recommendations by a positive nasopharyngeal swab PCR test and/or chest CT scan suggestive of COVID-19 whether the patient was symptomatic or not.

Patients without a prior diagnosis of CLD, including those with abnormal liver function tests or cytolytic hepatitis in the setting of SARS-CoV-2 infection, were excluded from the registry.

Organ transplant recipients were included prospectively in our registry from October 2020 and retrospectively using the liver transplant database from the French Society of Transplantation (SFT), which identified cases during the first wave of the epidemic (April 2020-September 2020). Inclusion criteria for the SFT database were i) symptomatic patients over 18 years of age; ii) history of solid organ transplantation (liver, kidney, heart or bi-organ); iii) infection with SARS-CoV-2 diagnosed by PCR and typical imaging. All transplanted recipients other than liver graft (n = 13) had an associated CLD.

Immunosuppression was defined as a history of organ transplantation, immunosuppressive treatment, HIV infection regardless of CD4 count, hematologic malignancy or extrahepatic solid cancer that was under treatment.

A dedicated e-CRF created by AFEF in May 2020 on the CleanWeb platform entitled"COVID and Liver"was accessible to all investigators (physicians or clinical research associates from hospitals, university hospitals, clinics) who requested an access code from the clinical research unit of Saint Louis Fernand Vidal Lariboisière. The medical information systems program was used in some centers to optimize the inclusion of hospitalized patients by cross-referencing the"COVID-19"code with different diagnostic codes for liver diseases from the International Classification of Diseases version 10 (ICD-10).

Baseline variables recorded prior to SARS CoV-2 infection included demographic, clinical, and laboratory characteristics related to etiology and severity of underlying CLD.

Advanced liver fibrosis (F) included Metavir F3 and F4 liver fibrosis stages. According to Baveno 7, compensated advanced liver disease reflecting the continuum of severe fibrosis and cirrhosis (F3/4) was defined by each investigator either on the basis of liver biopsy if ever performed or on the basis of liver stiffness measurement (LSM) values above 10 kPa (suggestive) or 15 kPa (very suggestive)^14^.

In addition, we collected clinical and biological variables at the time of SARS-CoV-2 infection diagnosis and during follow-up.

The date of the last visit was recorded, as were the date and cause of death.

### Statistical analyses

The overall population was split in distinct 2 groups: transplanted recipients (n = 271), including mainly liver transplants (n = 258, 95.2%) with no longer CLD in almost all the cases at the time of inclusion in the cohort, and non-transplanted patients (n = 948).

Moreover, within the non-transplanted population, different subgroups were considered, including advanced CLD subgroup and compensated CLD, defined as either non-advanced fibrosis (F0–F2) or advanced fibrosis (F3/F4) with preserved liver function (Child–Pugh A).

Categorical variables are presented as the number (%) of patients with the characteristic of interest, while continuous data are reported as mean ± standard deviation or median with interquartile range (IQR), as appropriate. The Mann–Whitney U test was used to compare non-normally distributed continuous variables between two groups. Categorical variables were compared using Pearson’s chi-squared test or Fisher’s exact test, as appropriate. The primary outcome was short-term (30-day) mortality.

To explore associations between selected variables (age, sex, body mass index, mild fibrosis, Child–Pugh score, significant cardiovascular history, advanced liver fibrosis, and diabetes mellitus) and 30-day mortality, univariate and stepwise multivariate Cox proportional hazards models were constructed. The proportional hazards assumption was tested using Schoenfeld residuals for both univariate and multivariate models. The results are reported as hazard ratios (HRs) with 95% confidence intervals (CIs). Time-to-event data were analyzed using Kaplan–Meier survival curves and compared using the log-rank test. Missing data were handled using multiple imputation techniques. All statistical analyses were performed with a two-sided significance level of 5% using SAS software version 9.4.

## Results

### Global population description

Between August 5, 2020, and December 31, 2021, 1219 adults were included from 30 different hospitals (24 university, 5 General public, 1 private) from all over France. Seventy-nine percent of the patients were included from 10 centers, 9 of which were university hospitals. Among the 1219 enrolled patients, 1132 (93%) were input directly into the registry, and 87 were included from the SFT transplant database. The main baseline characteristics of patients prior to SARS-CoV-2 infection are described in Table 1. Males represented 61.9% of the population (754 patients), with a median age of 61 years and frequent comorbidities (diabetes in 313 patients (25.7%), cardiovascular history in 248 patients (20.3%), obesity in 257 patients (21.1%), and dialysis renal failure in 26 patients (2.1%)).

**Table 1:** Description of populations : global population (n = 1219), non-transplant population (n = 948) and transplant population (n = 271).

	Available data n (%)	Global population n (% of available data) or median n = 1219	Available data n (%)	Non-transplant population n (% of available data) or median (IQR) n = 948	Available data n (%)	Transplant population n (% of available data) or median (IQR) n = 271
Male	1028 (84.3)	754 (73.3)	764 (80.59)	473 (61.9)	264 (97.42)	181 (68.6)
Age (years)	1036 (85)	61.0 (49.0 −69.0)	770 (81.22)	61.0 (49.0–70.0)	266 (98.15)	62.0 (52.6–69.0)
Tobacco use (active)	911 (74.7)	127 (13.4)	668 (70.46)	101 (15.1)	243 (89.67)	26 (10.7)
Alcohol use (active)	241 (19.8)	100 (41.5)	223 (23.52)	97 (43.4)	18 (6.64)	2 (11.1)
**Comorbidities**						
Immunosuppression	1047 (85.9)	366 (34.9)	776 (81.9)	95 (12.2)	271 (100)	271 (100)
Organ transplant	1047 (85.9)	271 (25.9)	776 (81.9)	0	271 (100)	271 (100)
Diabetes mellitus	1040 (85.3)	313 (30.1)	775 (81.8)	206 (26.6)	265 (97.8)	106 (40)
Cardiovascular events (HBP^1^/stroke/coronaropathy)	1037 (85.1)	248 (23.9)	775 (81.8)	153 (19.7)	262 (96.7)	95 (36.3)
BMI^2^ > 30 kg/m^2^	893 (73.3)	257 (28.8)	717 (75.6)	185 (25.8)	176 (64.9)	54 (30.7)
Chronic respiratory failure	1037 (85.1)	60 (5.8)	773 (81.5)	41 (5.3)	264 (97.4)	19 (7.2)
Dialysis renal failure	951 (78)	26 (2.7)	775 (81.8)	16 (2.1)	176 (64.9)	10 (5.7)
Liver fibrosis	985 (80.8)		745 (78.6)			NA*
F0/F1/F2		557 (56.4)		305 (40.9)		
F3/F4		477 (48.4)		440 (59.1)		
Child Pugh (for 440 patients with advanced liver fibrosis)	404 (95.1)		392 (92.2)			NA*
Grade A		239 (59.2)		228 (58.2)		
Grade B		123 (30.4)		122 (31.1)		
Grade C		42 (10.4)		42 (10.7)		
Etiologies	1047 (85.9)		776 (81.9)			NA*
Alcohol (active or weaned)		161 (15.4)		145 (18.7)		
Viruses		188 (17.9)		169 (21.8)		
MASLD		154 (14.7)		123 (15.9)		
Combined (alcohol, MASLD, viruses)		165 (15.8)		141 (18.2)		
Others		379 (36.2)		198 (25.1)		
Primary Liver tumors						NA*
HCC (active or cured)	952 (78.1)	184 (19.3)	773 (81.5)	138 (17.9)		
Cholangiocarcinoma (active or cured)	949 (77.9)	13 (1.4)	770 (81.2)	13 (1.7)		

NA*: not applicable because liver transplant recipients no longer had liver disease.

### Description of underlying liver disease into the non-transplant population (Table 1)

305 patients (40.9%) had no to mild fibrosis (F0, F1, F2), and 440 (59.1%) had advanced liver fibrosis, defining by a degree of fibrosis classified F3 or F4, among whom 164 had decompensated CLD (Child Pugh B or C). The degree of fibrosis was assessed by non-invasive tests in 576 patients (47.3%) and/or histology in 312 patients (25.6%).

Among the 440 patients with advanced liver fibrosis, 228 were Child‒Pugh A (58.2%), 122 Child‒Pugh B (31.1%), and 42 Child‒Pugh C (10.7%). The median MELD score was 9 (IQR 5–15).

The reported etiologies of CLD were viral infection (active or cured Hepatitis C and active or inactive Hepatitis C) in 169 patients (21.8%), past or current excessive alcohol consumption (OH) in 145 patients (18.7%), Metabolic-dysfunction Associated Steatotic Liver Disease (MASLD) in 123 patients (15.9%), mixed (association MASLD ± OH ± virus) (141, 18.2%) or other (autoimmune chronic hepatitis, biliary disease, hemochromatosis, porto-sinusoidal liver disease) (n = 198, 25.1%).

A primary malignant liver tumor (current or past) was recorded in 151 patients (19.6%), mostly hepatocellular carcinoma (HCC) (n = 138) and more rarely cholangiocarcinoma (n = 13).

### Description of the immunosuppressed patients

Globally, 34.9% of the 1219 enrolled patients (n = 366) were immunocompromised, including mostly 271 (25.9%) organ transplant recipients (among them 258 liver transplants), and also 60 patients with treated autoimmune hepatitis (5.7%), 10 patients with HIV infection (0.9%), and/or 11 patients (1.1%) with treated hematologic malignancies. Among 316 patients (25.9%) with declared immunosuppressive treatments, 194 received tacrolimus, 77 systemic corticosteroids, 35 azathioprine and 23 others drugs including anti-TNFα, chemotherapy and Rituximab.

### Description of the transplant population (Table 1)

258 (95.2%) out of 271 transplanted patients had received liver graft with no longer CLD in almost all the cases at the time of inclusion in the cohort.

Compared to the nontransplant population, transplanted recipients were less often female, older, with less consumption of tobacco and alcohol. They were more frequently obese and more often with diabetes mellitus and cardiovascular events history.

### Description of COVID-19 characteristics (Table 2)

**Table 2:** COVID diagnosis criteria, medical care and liver complications at enrollment.

	Available data	Overall population (n = 1219)
	N (%)	N (% of available data)
**Symptoms**	1188 (97.5)	772 (64.9)
Cough/expectorations		388 (32.7)
Fever/chills		505 (42.5)
Dyspnea/chest pain		313 (26.3)
**Systematic screening before treatment/contact**	1200 (98.4)	391 (32.6)
**Diagnostic methods**	1204 (98.8)	
Nasal swabbing PCR positive		976 (81.1)
Thoracic CT-scan in favor		340 (28.2)
Both PCR positive and Thoracic CT in favor		313 (25.9)
**Medical care**		
Hospitalization	1217 (99.8)	514 (42.2)
Intensive care unit	1186 (97.3)	128 (10.8)
Medecine unit	1186 (97.3)	355 (29.9)
**Liver complications at enrollment**	1197 (98.2)	
Acute cytolytic hepatitis (ALAT > 10ULN)		36 (3)
Ascitis		75 (6.3)
Encephalopathy		73 (6.1)

SARS-CoV-2 infection was diagnosed by positive nasal PCR in 80.1% of cases (976 patients), or a suggestive chest CT scan in 27.9% of cases (340 patients), among whom patients had both modalities in 25.7% of cases (313 patients).

Patients were symptomatic in 63.3% of cases. The diagnosis was incidental in the context of contact or systematic screening before a medical procedure or visit in 32.1% of cases.

At the time of diagnosis of SARS-CoV-2 infection, acute cytolytic hepatitis (liver enzymes > 10 times the upper limit of normal (ULN)) was recorded in 36 patients (2.9%), without acute liver failure reported (fulminant hepatitis). Ascites decompensation was reported in 75 patients (6.2%) and encephalopathy was reported in 73 patients (6%).

Within the limits of the eCRF information and retrospective data collection, immunosuppressive therapy would have been modified or stopped in 60 patients (16% of the immunosuppressed).

### Outcome and short-term mortality

**Fig. 1:**
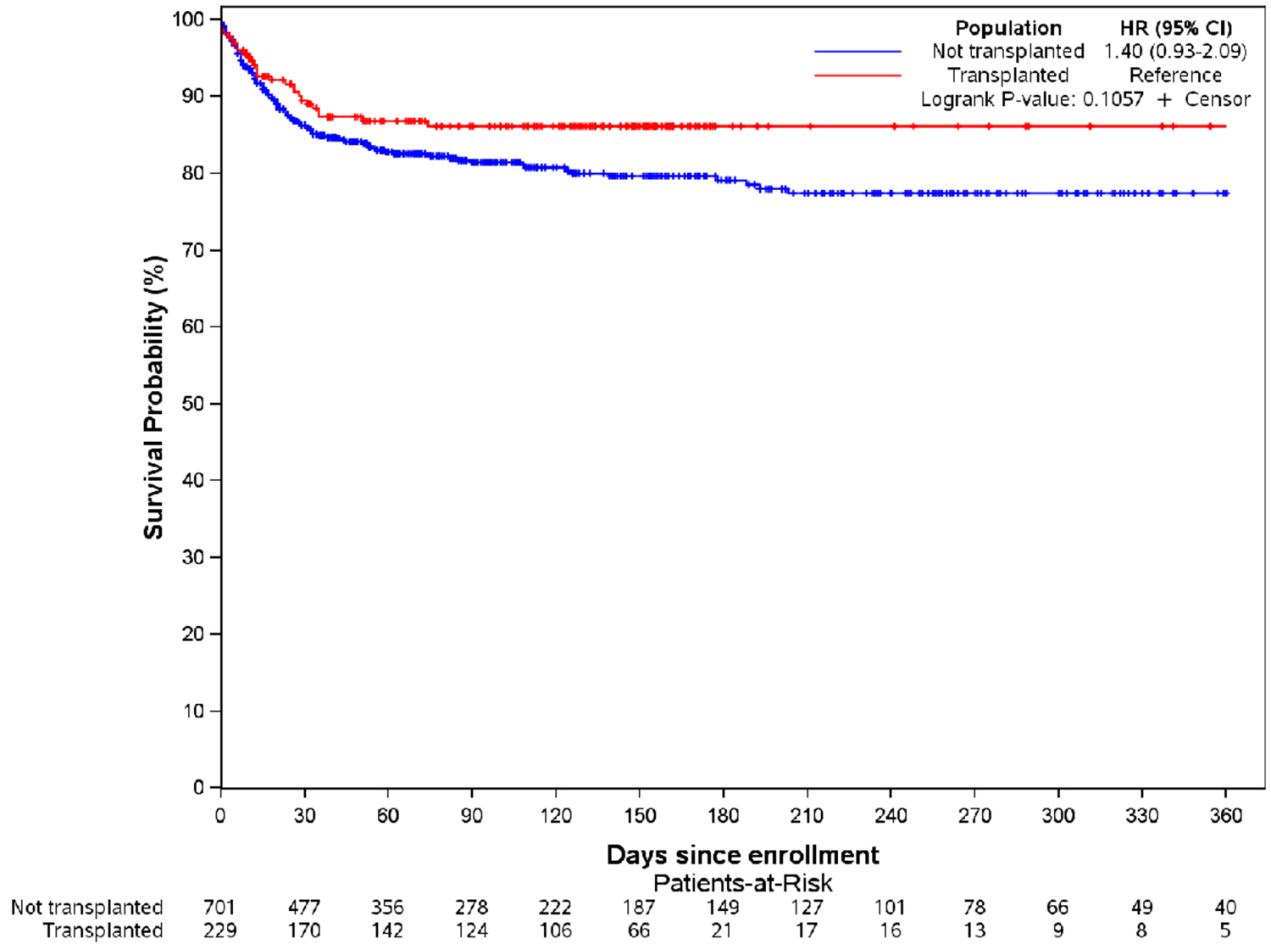
Kaplan Meier curve of the transplant population (11.1%, 30 deaths censored during a median follow up of 108 days) and the non-transplant population (16%, 124 deaths censored during a median follow-up of 61 days) (p = 0.106).

In the overall cohort, the global 30-day mortality was 13% (159 deaths censored) and the median follow-up time was 68 days (IQR 21–154 days). Moreover, no significant difference in 30-day mortality was observed in the transplant population (11.1%, 30 deaths censored, median follow up of 108 days) compared to the non-transplant population (30-day mortality 16%, 124 deaths censored, median follow-up of 61 days (Fig. 1).

Most of the deaths were non-liver-related (n = 99, 62.3%). A minority of patients (n = 48, 30.2%) died from hepatic causes and data were missing for 12 dead patients.

The median time from inclusion in the observatory to death was 15 days (IQR 6–31 days).

A total of 514 patients required hospitalization (42.2%), including 128 in intensive care (10.5%) among whom, 54 patients were had advanced liver fibrosis (42.2%).

The majority of patients remained at home or returned home (82.8%) and 51 patients were still hospitalized at the time of inclusion (4%) (Table 2).

### Prognostic factors for short-term mortality

In the overall *non-transplant population*, factors associated with 30-days mortality identified by univariate analysis included: male sex (HR 2.066, p = 0.0006), age (HR 1.041, p < 0.0001), diabetes mellitus (HR 2.516, p < 0.0001), cardiovascular events (HR 2.02, p = 0.0003), obesity (HR 1.083, p = 0.0184) chronic respiratory failure (HR 2.272, p = 0.0052), advanced liver fibrosis (HR 7.356, p < 0.0001), alcohol-related etiology (alone or mixed) (HR 4.333, p < 0.0001), Child–Pugh class B or C (HR 7.738, p < 0.0001), history of HCC (HR 2.103, p = 0.0002) or cholangiocarcinoma (HR 3.119, p = 0.013) (Table 3).

**Table 3:** Univariate analysis on non transplant population (n = 948) and transplant population (n = 271).

	Non transplant population n = 948		Transplant population n = 271	
	**HR (IC95%)**	**p**	**HR (IC95%)**	**p**
Male sex	**2.066**	**0.0006**	1.564	0.301
Age	**1.041**	** <.0001**	**1.082**	** < .0001**
Immunosuppression	0.599	0.121	NA^1^	
Diabetes mellitus	**2.516**	** <.0001**	1.896	0.081
Cardiovascular events	**2.02**	**0.0003**	1.645	0.176
BMI > 30 kg/m^2^	**1.083**	**0.0184**	1.272	0.618
Chronic respiratory failure	**2.272**	**0.0052**	**3.209**	**0.012**
Dialysis renal failure	1.094	0.88	**4.732**	**0.023**
Liver Fibrosis F0-1–2 vs F3-4	**7.356**	** <.0001**	NA*	
Initial Child Pugh grade B/C vs A (for advanced liver fibrosis patients)	**7.638**	** <.0001**	NA*	
Alcohol related liver disease (pure or combined)	**4.333**	** <.0001**	NA**	
HCC active or cured	**2.103**	**0.0002**	NA***	
Cholangiocarcinoma active or cured	**3.119**	**0.013**	NA***	

^1^100% patients in this population were indeed immunosuppressed, because transplanted patients.

*liver transplant recipients no longer have liver disease/liver fibrosis at time of inclusion (except for 13 other than liver recipients).

**liver transplant recipients no longer have causes of chronic liver disease at time of inclusion (except for 13 other than liver recipients).

***liver transplant recipients no longer have primary liver cancer at time of inclusion.

Immunocompromised status was not significantly associated with increased short-term mortality in univariate analysis and may have had a protective trend (HR 0.599), also this was not statistically significant (p = 0.121) (Table 3).

Independent prognostic factors associated with 30-day mortality were advanced liver fibrosis (HR 2.537, p = 0.007), obesity (HR 1.556, p = 0.038) and age (HR 1.038, p < 0.0001) (Table 4).

**Table 4:** Multivariate analysis with Cox Model In overall non-transplant population (a), in non-transplant patients with advanced liver fibrosis (b) and subgroup of non-transplant patients with compensated Chronic Liver Disease (CLD), defined as either non-advanced fibrosis (F0–F2) or advanced fibrosis (F3/F4) with preserved liver function (Child–Pugh A) (c).

Independant Factors of 30-day mortality	p	HR			95% CI			
**a. Overall non- transplant population (n = 948)**								
Fibrosis (advanced F3-4 vs F0-1–2)	0.007	2.537			1.297		4.965	
Body Mass Index > 30 kg/m^2^	0.038	1.556			1.025		2.362	
Age	<.0001	1.038			1.019		1.057	
**b. Non- transplant population with advanced liver fibrosis (n = 440)**								
Child–Pugh B/C vs A	<.0001		3.872	0.167				0.399
Male sex	0.019		1.79	1.101				2.908
Age	<.0001		1.04	1.02				1.061
**c. Non- transplant population with compensated CLD, defined as either non-advanced fibrosis (F0–F2) or advanced fibrosis (F3/F4) with preserved liver function (Child–Pugh A) (n = 533)**								
Advanced fibrosis (F3-4 vs F0-1–2)	0.003	3.486			1.55	7.839		
Diabetes mellitus	0.014	2.273			1.179	4.382		
Age	0.009	1.041			1.01	1.072		

In the subgroup of non-transplant patients with advanced liver fibrosis (n = 440), the independent prognostic factors associated with 30-day mortality were Child Pugh B/C (HR 3.872, p < 0.0001), male sex (HR 1.79, p = 0.019) and age (HR 1.04, p < 0.0001) (Table 4). Finally, in patients with compensated CLD, defined as either non-advanced fibrosis (F0–F2) or advanced fibrosis (F3/F4) with preserved liver function (Child–Pugh A) (n = 533), short-term mortality was driven by advanced liver fibrosis (HR 3.486, p = 0.003), diabetes mellitus (HR 2.273, p = 0.014 and age (HR 1.041, p = 0.009) (Table 4).

In the transplant population, while univariate analysis identified dialysis renal failure (HR 4.732, p = 0.023), chronic respiratory failure (HR 3.209, p = 0.012) and age (HR 1.082, p < 0.0001), as factors significantly associated with 30-days mortality (Table 3), age remained the only independent prognostic factor (HR 1.177, p 0.003) in multivariate analysis.

**Fig. 2:**
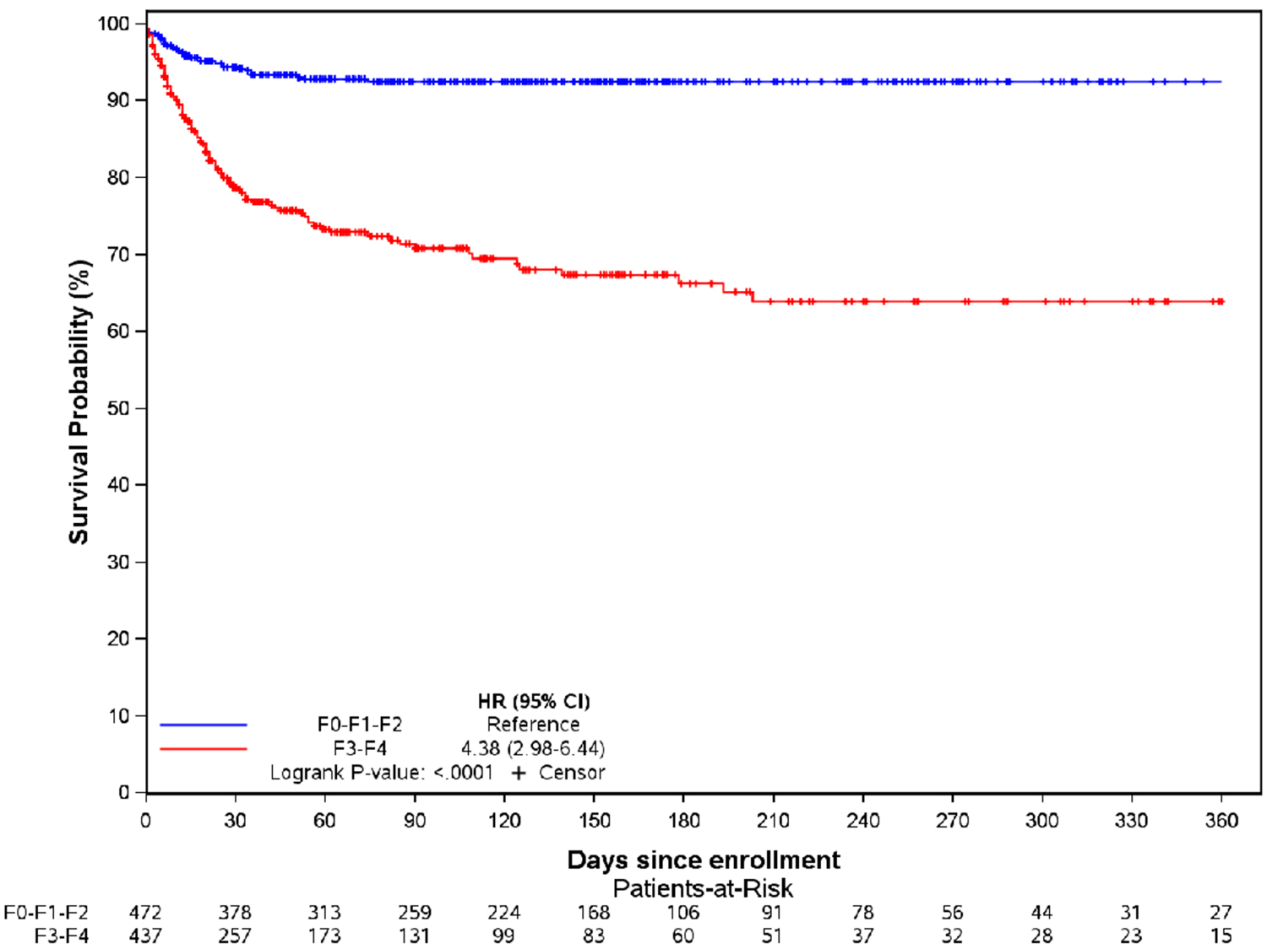
Kaplan Meier curve according to liver fibrosis stages (F0-F1-F2 vs. F3-F4) in the overall non-transplant population (n = 948, p < 0.0001).

**Fig. 3:**
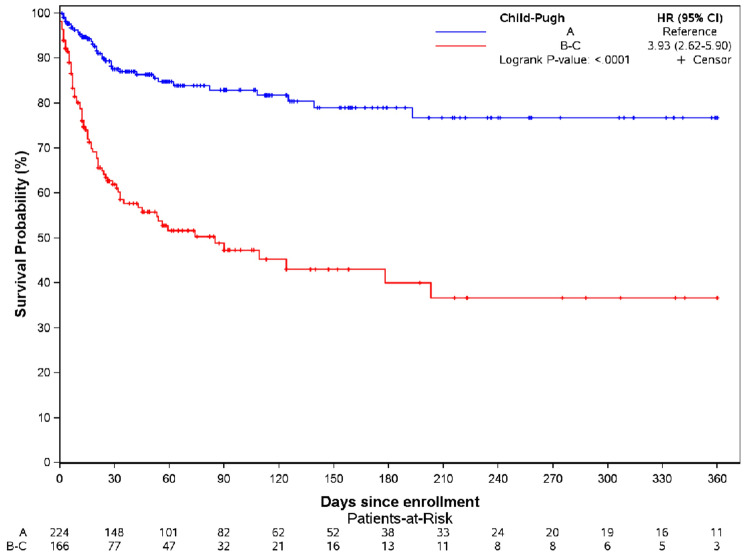
Kaplan Meier curve according to the Child‒Pugh score (A vs. B-C) in the non-transplant population with advanced liver fibrosis (n = 440).

Figures 2 and 3 illustrate the prognostic impact of advanced liver fibrosis and decompensated CLD, with significant difference of 30-day mortality according to liver fibrosis stages (F0-F1-F2 vs F3-F4) in the overall non-transplant population (p < 0.0001) (Fig. 2) and according to Child‒Pugh score in the subgroup of patients with advanced liver fibrosis (p < 0.0001) (Fig. 3).

## Discussion

The present registry is the only French multicenter “real life” cohort, mainly prospective, and the largest one, describing 1219 SARS-CoV-2-infected patients with underlying CLD. Indeed, other international registries have been reported, the largest of which, reported by Marjot et al., included 745 patients followed for CLD but excluded liver transplant patients^3^. Other concordant results come from public health databases^6,15–18^ including a French retrospective study^6^ based on Hospital Discharge database without detailed clinical characteristics of the patients.

The primary outcome confirms the major prognostic impact of advanced liver fibrosis on COVID-19-related short-term mortality. In particular, the results confirm the excess risk of death induced by the presence of advanced liver fibrosis, in both compensated advanced CLD and a fortiori in decompensated CLD population. Thus, advanced fibrosis was the main independent prognostic factor in this observational study, with an HR = 2.273, regardless of the Child‒Pugh score. Age was also a predictive factor in the entire population, wether in transplanted patients or non-transplanted patients.

The mortality in non-transplant population with CLD was 16% in our cohort, that is lower than in other previous registries and cohorts that also showed an excess mortality of patients with cirrhosis, but of approximately 30%^3,15,16^. This difference could be explained by the low rate of patients with decompensated liver function in our population.

In the French national hospital discharge database report by Mallet et al.^6^, it seemed that the reduced access to intensive care and mechanical ventilation might have resulted in the excess mortality of patients with cirrhosis but this information was not collected in our study.

Nevertheless, according to Brozat et al.^15^, the excess mortality of cirrhotic patients could be explained in part by the frequent comorbidities in these patients (diabetes, obesity, hypertension, chronic renal failure, etc.). In our study, only obesity was associated with mortality in multivariate analysis (HR 1.556).

On the other hand, a Swedish study of a population of patients with CLD showed that the presence of CLD was associated with an increased risk of hospitalization for COVID-19, but COVID-19-related mortality was not increased^17^. This result was a key argument behind our French liver society convincing the national health authorities to prioritize vaccination of patients with compensated cirrhosis in March 2021 before vaccination became universal.

In our population, we observed acute cytolytic hepatitis (defined by ALT > 10 ULN), but no acute liver failure related to COVID-19, while there are cases reported in a German cohort of 147 patients with liver injury in SARS-CoV-2 infection, but without pre-existing liver disease and risk factors associated were young age, obesity and male sex^19^.

The other major finding of the study was the absence of excess mortality risk in the subpopulation of immunocompromised patients, particularly in the subgroup of transplant patients, which represented 22% of the overall population, probably because, after transplant, the recipient has a normal and functional liver. This study is the largest cohort of liver transplant recipients with COVID-19 and confirms the results of several studies on liver transplant patients^10,11,20^, in contrast to the results on kidney transplant patients, which have been associated with an increase in mortality related to COVID-19, probably because of their numerous associated comorbidities (chronic kidney failure, cardiovascular, type 2 diabetes, arterial hypertension, advanced age) and their high level of immunosuppression. There was no excess mortality in patients followed for autoimmune hepatitis, confirming the data of Marjot et al.^21^.

Regarding the etiology of CLD, we found alcohol to be a factor associated with excess mortality in univariate analysis, as did the registry studies of Marjot et al. and the French database from Mallet et al.^3,6^ but this was not confirmed in multivariate analysis in our study.

Other studies have shown that the prevalence of MASLD ranges from 28 to 37% of patients infected with SARS-CoV-2^15^. MASLD was associated with greater severity of COVID-19^22^, particularly in the subgroup of patients under 65 years of age in the meta-analysis by Wang et al.^13^, and with increased mortality in these patients, with an OR of 2.93 (95% CI 1.87–4.6)^23^, though there were no details on the degree of liver fibrosis.

The presence of a primary liver tumor was a collected data, but was associated with mortality in our study only in univariate analysis, and not confirmed in multivariate analysis. Some studies have investigated the impact of COVID-19 on the management and prognosis of HCC, one large international study showing a change in management (screening, diagnosis or treatment) in 80% of cases^24^. In a French study of 670 patients (in two periods, 2019 vs. 2020), the number of new HCCs presented to the multidisciplinary consultation meeting was lower during the COVID-19 period, and the time to treatment was extended by one month in 20% of patients^25^. More generally, the number of patients newly treated for digestive cancer, especially for HCC, fell drastically during the lockdown period (−42%) in a large population of patients over 65 years of age^26^.

Our study has several limitations. The registry is not exhaustive in part because the inclusions were nonconsecutive and made by voluntary investigators, thus leading to a selection bias. Moreover, the observational was partially retrospective and was of a declarative nature, resulting in a large number of missing data, particularly biological data, which must be considered in interpreting the results. But it is worth noting that the number of missing data was less than 20% in the majority of the cases, particularly for major items such as immunosuppressed status, degree of fibrosis and etiology of liver disease. However, to prevent interpretative bias due to missing data, we used multiple imputation techniques.

Thus, although data from several large studies, including a meta-analysis of 64 studies involving more than 11,000 patients, showed that abnormalities on liver tests, mainly cytolysis, were frequent (> 20% of infected patients)^9^ and were associated with excess mortality in hospitalized infected patients^8^, in our study the biochemical data and the prognostic effect of disturbances of liver homeostasis were not interpretable.

We did not collect information regarding the occurrence of post-COVID-19 cholangitis in the follow-up of patients, as there were too many missing biological data. This complication of chronic post-COVID-19 cholangitis has been regularly reported in the literature^27–29^. The pathophysiology could be multifactorial, combining at least a direct viral toxicity, ischemic cholangitis similar to resuscitation cholangiopathies and a drug toxicity, in particular from ketamine.

Finally, as the observation was initiated before the start of universal vaccination programs against SARS-CoV-2, information on the vaccination status of the patients was not available. In addition, given the inclusion period, a minority of patients had an optimal vaccination schedule as currently recommended.

## Conclusion

In the present large cohort of 1219 patients who were previously followed for CLD or after organ transplantation (mainly liver) and infected with SARS-CoV-2, the overall 30-day mortality was 13% (11% in transplant population and 16% in non-transplant population). In the non-transplant group, the presence of advanced fibrosis was associated with excess mortality whether or not liver function was impaired. conversely, immunocompromised status, was not associated with excess mortality. Our registry results are consistent with the management guidelines recently issued by the EASL^30^.

## Data Availability

The data used to support the findings of this study are included within the article.

## Abbreviations

AFEF: Association Française de l’Etude du Foie / French Liver Society
EASL: European Association for the Study of the Liver
CLD: Chronic Liver Disase
COVID-19: Coronavirus SARS-Cov2 related Disease
CT scan: Computed Tomography scan
F: Liver Fibrosis
HCC: Hepatocellular carcinoma
HR: Hazard Ratio
MASLD: Metabolic-dysfunction Associated Steatotic Liver Disease
OH: Alcohol
PCR: Polymerase Chain Reaction
SARS-CoV-2: Severe acute respiratory syndrome coronavirus 2
SFT: Société Francophone de Transplantation / French speaking Society of Transplantation
ULN: Upper Limit of Normal
